# Male Gynecomastia Correction by Superior Dynamic Flap Method: A Consistent and Versatile Technique

**DOI:** 10.29252/wjps.9.1.33

**Published:** 2020-01

**Authors:** Sreekar Harinatha

**Affiliations:** Department of Plastic and Cosmetic Surgery, Contura Clinic, Bangalore, India

**Keywords:** Gynecomastia, Male, Breast, Flap

## Abstract

**BACKGROUND:**

Gynecomastia is a common cosmetic issue in India. Many combinations of liposuction and excision of the gland have been advocated. Complete removal of the breast leaves behind a contour deformity and hence a sliver of it needs to be left behind. This study used a consistent and versatile technique for correction of gynecomastia by a superior dynamic flap method.

**METHODS:**

A retrospective study was conducted in 1159 gynecomastia surgeries done from March 2013 to February 2019. All these patients were treated by a single surgeon using the superior dynamic flap method in a single center and the results were assessed with patient and surgeon satisfaction scores. The technique involved leaving behind a thin sliver of the gland in order to avoid contour deformity and achieve a smoother shape overall.

**RESULTS:**

Minor complications were seen in 27 patients and the satisfaction scores were 8.9 and 8.79 in patients and surgeon, respectively. There was no incidence of contour deformity after the procedure.

**CONCLUSION:**

Superior dynamic flap method was a versatile technique and allowed the surgeon not only to avoid contour deformities, but also to correct asymmetries.

## INTRODUCTION

Gynecomastia is defined clinically as an enlargement of male breast tissue. It is characterized by the presence of a rubbery or firm mass extending in a diffuse and concentric fashion under the nipple and areola. Histopathologically, it is characterized by a benign proliferation of glandular male breast tissue not unlike of that in females.^1^ It can be physically uncomfortable, psychologically distressing and may have a negative impact on the self-confidence and individual’s overall body image. Pseudo-gynecomastia is common in obese men, and consists of lipomastia alone, without glandular proliferation and is a common differential diagnosis.^2^

True gynecomastia occurs bilaterally in most cases and is the most common breast condition in males.^3^ The incidence is on the rise due to usage of various medical drugs, recreational drug abuse and environmental contamination.^4^ It is estimated that 30-60% of boys exhibit gynecomastia during adolescence and that at least 30-50% have some amount of breast tissue. A trimodal age wise distribution pattern is observed in the incidence of gynecomastia. The first peak is in the neonates, followed by puberty and lastly in old males. In each of these peaks, the cause is an altered hormonal status.^5^

In mild cases, simple reassurance coupled with advice on diet and exercise may be sufficient; though surgery may be needed in those affected by it psychologically. In severe cases, medical and/or surgical intervention is required.^6^^,^^7^ Our technique of gynecomastia correction was based on the preservation of a thin sliver of the glandular tissue in order to achieve a better aesthetic appearance of the chest.

## MATERIALS AND METHODS

A retrospective study was conducted of all the 1159 gynecomastia surgeries done from March 2013 to February 2019. All these patients were treated by a single surgeon using the superior dynamic flap method in a single center. The physical examination included evaluation of height and weight, examination of the breasts, genitals, liver, body hair patterns, lymph nodes, and thyroid. Assessment of symmetry and consistency of breast tissue was done. In a majority of cases, the gynecomastia was bilateral, although unilateral symptoms did occur. The amount of asymmetry in bilateral cases was noted and marked on the evaluation sheet. Also, important was to rule out pseudo-gynecomastia by assessing consistency of the sub-areolar tissue. In our clinic, we noted down the examination findings on a sheet including the demographic and clinical information.

Though Simon’s grading is simple and used more widely, one additional factor that we assessed was the skin tone. To put it simply, while skin excess is the loose skin on top of the breast tissue, skin tone is the inherent capacity of the skin to shrink and contract after the surgery. While skin excess generally has a linear progression from grades 1 to 3, skin tone can be independent of it. This often neglected factor can be the difference between a well sculpted chest and an average result. We assessed skin tone by pinching the skin over the chest and releasing and observing the speed at which it snapped back. Though this is fairly subjective, it does give one an idea about the overall skin tone.

The revised grading was as follows: Grade IT: Small enlargement, No skin excess, Normal skin tone, Grade 1L: Small enlargement, No skin excess, Poor skin tone, Grade IIAT: Moderate enlargement, No skin excess, Normal skin tone, Grade IIAL: Moderate enlargement, No skin excess, Poor skin tone, Grade IIBT: Moderate enlargement, Minimal Skin excess, Normal skin tone, Grade IIBL: Moderate enlargement, Minimal Skin excess, Poor skin tone, Grade IIIT: Marked enlargement, Lot of excess skin, Normal skin tone, and Grade IIIL: Marked enlargement, lots of excess skin, poor skin tone. In most cases gynecomastia is a purely cosmetic issue and the targets of correction are (i) Achieve an aesthetic chest, (ii) Achieve symmetry between either sides, and (iii) Minimize the risk of complications.

**Fig. 1:**
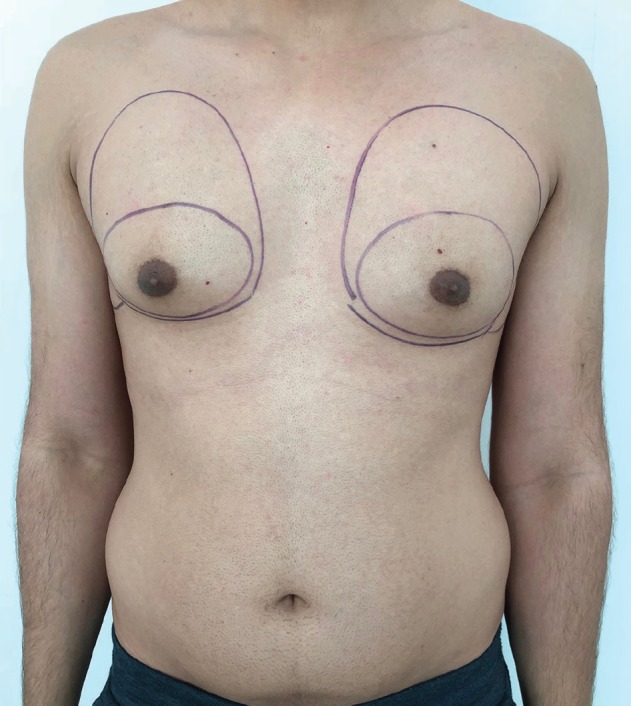
Pre-surgical markings making note of the asymmetry between the two sides. The outer marking would be the extent of liposuction and the inner marking the extent of breast tissue

The pre surgical markings were made in the preoperative holding area with the patient standing. The extent of the outer chest bulges were marked first. This mark also involved the area of excess fat that needed to be treated along with the breast tissue. This mark continued to the inframammary fold was marked. This mark formed the outer boundary of the surgical area. Then, the palpable gland was marked. To facilitate easier isolation of the gland, the pectoral muscles were contracted by asking the patient to press the hips with his arms. Once the muscle was taught, the gland stood out for an easier marking. Next, the glandular markings were confirmed by asking the patient to raise his arms while the breast tissue was held by the surgeon’s fingers. This was repeated on the other side. Also important to mark was the lateral chest wall folds of fat that also could be treated effectively for a better contour (Figure 1). 

The patient was positioned supine with the arms abducted and given general anesthesia. A hypotensive anesthesia was paramount to a bloodless field for dissection and for minimizing blood loss. First we started with liposuction through a stab incision at the inferior part of the areola and infiltrations of wetting solution performed in the “superwet” fashion. The infiltrate solution contained 1 ampoule of 1:1,000 adrenaline in 1L of lactated Ringer solution. Liposuction was performed with the conventional suction machine. The focus was on reducing the lateral fat folds, smoothening out the whole of the chest wall in accordance to the final result. The liposuction also helped in creation of an avascular dissecting field and isolated the gland to a greater extent making further dissection simpler. The aspirate volume was recorded and compared to the opposite side. The liposuction also proceeded through the gland onto the lateral areas. Once the liposuction was completed the gland dissection was started. 

An inferior peri-areolar incision was then made and dissection proceeds all around the 360 degrees. The initial plane of dissection was along the subcutaneous plane. Initially, the assistant raised the areola with a couple of skin hooks and the plane was dissected between the breast and the subcutaneous tissue. This extended all around until the skin with its fat was almost raised off the gland. Then the gland was elevated from the pectorals fascia starting inferiorly. The gland was elevated from all sides except for the superior aspect from where the vascularity remains intact.

Thus the gland was elevated as a superior based vascular flap. The gland was externalized and the amount of depression it created on the chest was assessed. Also the extent of any irregularities on the chest wall were assessed and marked. Then the gland was held taut and the excess gland was excised, whilst saving the necessary amount to compensate for the contour irregularities. This remaining gland normally was between 5-20% of the dissected tissue. The amount also was dependent on the overall patient contour, amount of fat, size of the pectoral muscles and the amount of asymmetry between the either sides that needed to be corrected. 

The remaining tissue was then inserted back through the incision and sutured to the pectorals major muscle with absorbable 3-0 sutures all around. As the vascularity of the glandular tissue was intact, the healing was usually smooth. Also the direction and extent of pull during fixation could be adjusted depending on the location of correction that was needed. Hemostasis was then checked and incision closed in layers with absorbable sutures. A compression dressing was done to close the dissection spaces and reduce the chances of seroma. No drains were used. A compression vest was advised for six weeks once the surgical dressing was removed after two days. Six weeks after the surgery, satisfaction was rated using a system similar to the visual analog scale from “0” to express no satisfaction and “10” for complete satisfaction that patient got the desired result from the surgery. The surgeon’s satisfaction rate was also assessed using the same rating.

## RESULTS

**Fig. 2:**
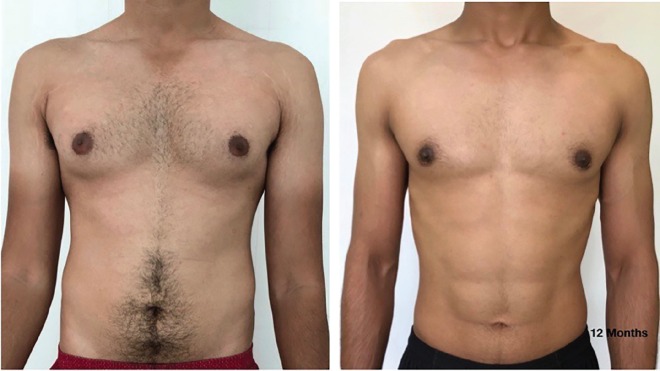
Pre and post-surgery (12 months) photos, grade 1T

**Fig. 3:**
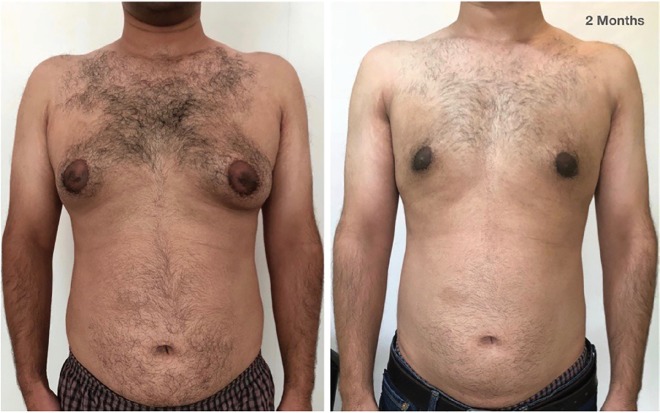
Pre and post-surgery (2 months) photos, grade IIAT on the right side and IIBT on the left

**Fig. 4:**
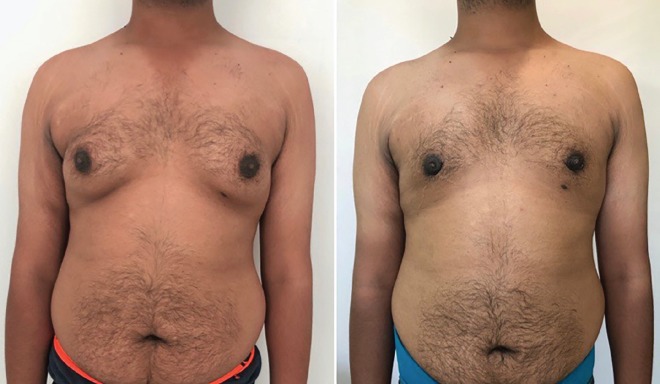
Pre and post-surgery (2 months) photos, bilateral grade IIAL

**Fig. 5:**
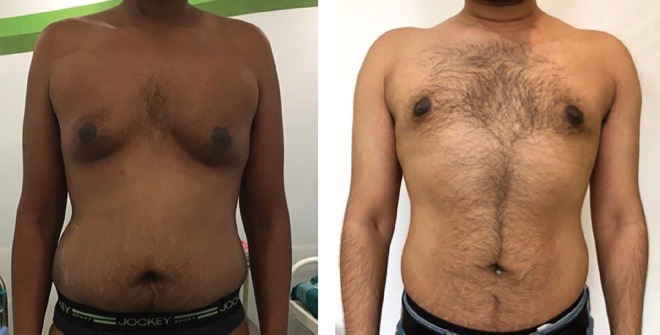
Pre and post-surgery (8 months) photos, bilateral grade IIBL

The grading of the 1159 cases were as follows: Grade IT: 366 cases (31.57%), Grade 1L: 3 (0.26%), Grade IIAT: 478 (41.24%), Grade IIAL: 17 (1.47%), Grade IIBT: 203 (17.52%), Grade IIBL: 18 (1.55%), Grade IIIT: 51 (4.4%), and Grade IIIL: 23 (1.73%). The complications seen were Hematoma/Seroma in 27 cases, Delayed wound healing in 32 and minor contour asymmetry in 7. There were no revision surgeries. The satisfaction rate was one of the main concerns of the study. The patients had a mean satisfaction score of 8.9±0.826 with the range of 5-10 from total 10 score (Figures 2-5). The majority of the patients had a score of 9. A similar rating was also used to assess the satisfaction of the surgeon. The surgeon’s score was 8.79±0.55. 

## DISCUSSION

Most men with gynecomastia feel the presence of a firm to hard mass under their nipples during the puberty. Most notice it under their school uniforms. Many of them are subjected to jokes or ridicule and some even suffer various psycho-social issues. Many students start wearing loose fitting shirts during these periods hoping the gynecomastia gets noticed less. Many others try working out. We have noticed many students in their high schools or early college working out endlessly in the gyms trying to burn the ‘fat’ under the nipples. They sometimes over-work enough to get disproportionately big chest muscles as compared to the rest of the body. Some extreme cases of steroid injections and unhealthy dietary supplements’ intake are not uncommon.

The first surgical treatment of gynecomastia is credited to Paulas Aegineta (625–690 AD), a Byzantine Greek physician.^8^ Webster in 1946 described semicircular intra-areolar incision. Current surgical techniques favor standard liposuction/suction-assisted lipectomy (SAL) and ultrasound-assisted liposuction (UAL) with a combination of removal of breast tissue, with the advantage of tackling both the fat and the breast tissue. Surgery should be considered in patients with cosmetic concern, discomfort, psychological stress and longstanding gynecomastia (>12 m).^9^

In general, all grades of gynecomastia are ideally treated with a combination of liposuction and excision (removal of the gland); Lipo-excision. This is especially true for grades I and II. In patients with Simon grade III, any form of liposuction may be combined with skin resection. The skin resection is usually delayed in most cases in darker individuals to wait for skin retraction and subsequently reduce the amount of scarring. The earlier gynecomastia surgeries concentrated on excising the breast tissue similar to a female mastectomy.^9^

A few unsatisfactory years later, there were many surgeons who advocated only liposuction for grades 1 and 2 of gynecomastia. On long term follow-ups, they noticed that the gland appeared like a projecting blob under the nipple and most patients were unsatisfied with the chest shape.^10^^-^^12^ They in fact felt that removing the fat worsened the chest, as the gland stood out even more without the fat to smoothen its edges a little. This is more true in Indian patients who have a more firmer breast tissue. This marked a paradigm shift in thinking and after a few more techniques came to fore, Teimourian and Pearlman described a combination of liposuction and removal of the gland for treating gynecomastia.^10^

It was called by various names and the commonest was ‘Lipo-excision’. Since then, a combination of liposuction and removal of the gland has been in vogue for treating gynecomastia. However, this seemingly simple combination was far from refined and has gone through its fair share of changes and experiments. The hot topic of debate was how one should balance between these two procedures. Many surgeons were happy leaving behind almost none of the gland, while others tended to leave more behind.^10^^-^^12^

There could be many reasons why there was such a gross difference in the techniques, though technically both were Lipo-excision. In most Indian cases where only excision was done, it almost always left behind a sunken contour deformity. Many surgeons advocate leaving behind a 2-3 cms disc of tissue under the nipple which is about 1cm thick. While this is justified in many cases, such small, but thick tissue may be unsuitable in many cases. We recommend leaving behind a wider disc sometimes extending even 10 cms in diameter, while the thickness can only be assessed intra-operatively.^10^^-^^12^

This is by no means a guideline and the extent and thickness of tissue to be left behind is best assessed by the surgeon, once he finishes isolating the gland. In a man with bulky chest muscles and less fat, the thickness of the gland left behind may be a few millimeters, which it may be thicker in people with more fat. This very customization depending on the patient’s builds and helps achieve a better symmetry and aesthetic appearance. Moreover, even in the same patient, the asymmetries can easily be corrected by leaving behind different size and amount of the gland to equate both the sides. Hence this method is aptly named the ‘superior dynamic flap method’.^10^^-^^12^

The other major advantage of this flap of glandular tissue is its robust vascularity. This allows the surgeon to mobilize the tissue and fix it in the areas, where it is needed. We have sometimes fixed the tissue on the lateral chest to achieve a better contour and because its vascularity is intact, the results are lasting and durable. While gynecomastia is a common issue in many men, the treatment options have been in a constant flux with a plethora a different surgical techniques described. Many techniques focus on different methods to remove the excess fat and more importantly the breast tissue. The use of superior dynamic flap method is a valuable tool in not only addressing the issues of contour deformities, but also to reduce the chances of asymmetry between the two sides.

## CONFLICT OF INTEREST

The authors declare no conflict of interest.

## References

[1] Rahmani S, Turton P, Shaaban A, Dall B (2011). Overview of gynecomastia in the modern era and the Leeds Gynaecomastia Investigation algorithm. Breast J.

[2] Johnson RE, Kermott CA, Murad MH (2011). Gynecomastia - evaluation and current treatment options. Ther Clin Risk Manag.

[3] Daniels IR, Layer GT (2001). Gynaecomastia. Eur J Surg.

[4] Barros AC, Sampaio Mde C (2012). Gynecomastia: physiopathology, evaluation and treatment. Sao Paulo Med J.

[5] Gikas P, Mokbel K (2007). Management of gynaecomastia: an update. Int J Clin Pract.

[6] Motamed S, Hassanpour SE, Moosavizadeh SM, Heidari A, Rouientan A, Nazemian M (2015). Successful Excision of Gynecomastia with Nipple Repositioning Technique Utilizing the Dermoglandular Flap. World J Plast Surg.

[7] Taheri AR, Farahvash MR, Fathi HR, Ghanbarzadeh K, Faridniya B (2016). The Satisfaction Rate among Patients and Surgeons after Periareolar Surgical Approach to Gynecomastia along with Liposuction. World J Plast Surg.

[8] Gurunluoglu R, Gurunluoglu A (2001). Paulus Aegineta, a seventh century encyclopedist and surgeon: his role in the history of plastic surgery. Plast Reconstr Surg.

[9] Kasielska A, Antoszewski B (2013). Surgical management of gynecomastia: an outcome analysis. Ann Plast Surg.

[10] Teimourian B, Perlman R (1983). Surgery for gynecomastia. Aesthetic Plast Surg.

[11] Fruhstorfer BH, Malata CM (2003). A systematic approach to the surgical treatment of gynaecomastia. Br J Plast Surg.

[12] Rosenberg GJ (1987). Gynecomastia: suction lipectomy as a contemporary solution. Plast Reconstr Surg.

